# Supplementary material for: Graphene wrinkling induced by monodisperse nanoparticles: facile control and quantification
Source: Sci Rep. 2015 Nov 4;5:15061. doi: 10.1038/srep15061 (PMC4632107; doi:10.1038/srep15061)
Supplement: Supplementary Information [file srep15061-s1.pdf]

Supplementary Information

Graphene wrinkling induced by monodisperse nanoparticles: facile control and quantification

Jana Vejpravova[1]*, Barbara Pacakova[1], Jan Endres[2], Alice Mantlikova[1], Tim Verhagen[1], Vaclav Vales[3], Otakar Frank[3] and Martin Kalbac[3]**

[1]Institute of Physics AS CR, v.v.i., Department of Magnetic Nanosystems, Na Slovance 2, 18221 Prague 2, Czech Republic

[2]Charles Univeristy in Prague, Faculty of Mathematics and Physics, Department of Condensed Matter Physics, Ke Karlovu 5, 12116 Prague 2, Czech Republic

[3]JH Institute of Physical Chemistry AS CR,v.v.i., Dolejskova 3, 18200 Prague 8, Czech Republic

*vejpravo@fzu.cz, **martin.kalbac@jh-inst.cas.cz

S.1. Additional results of Raman mapping

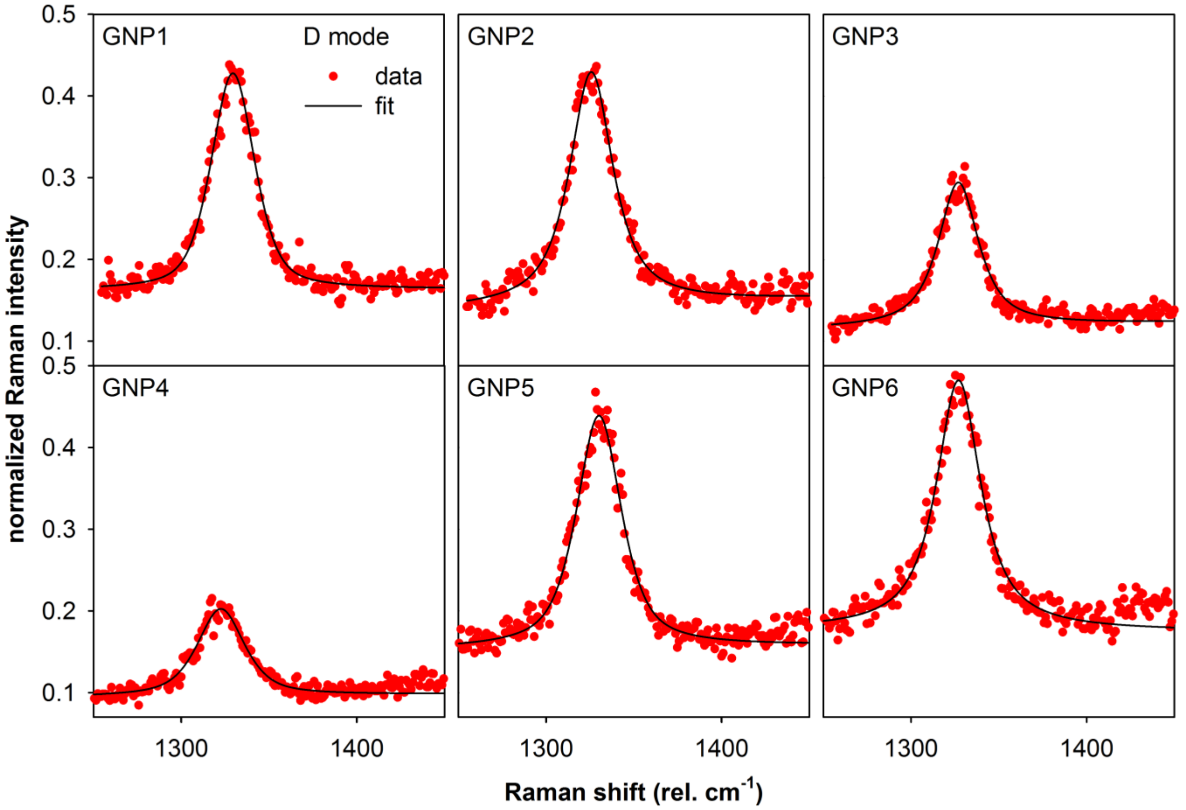

S1.1. Typical Raman spectra of the GNP1-6 samples in the D-mode region together of the fit by a single pseudo-Voigt function.

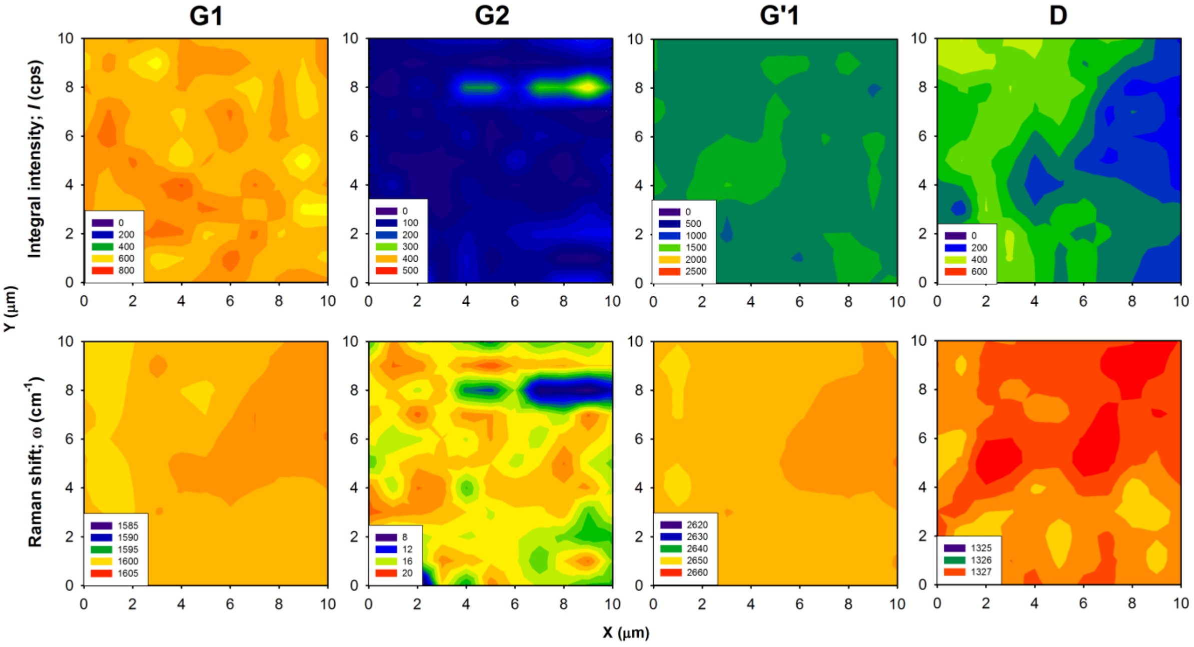

S1.2. Raman maps of Raman shift and integral intensity of the principal graphene modes for the GNP1 sample.

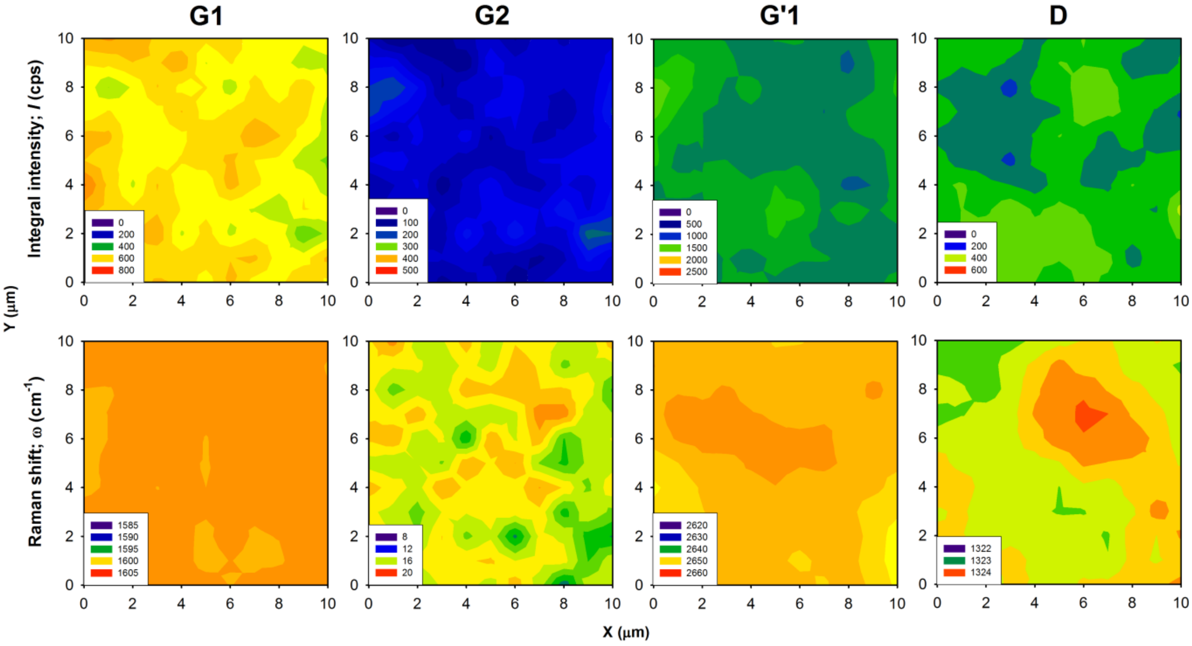

S1.3. Raman maps of Raman shift and integral intensity of the principal graphene modes for the GNP2 sample.

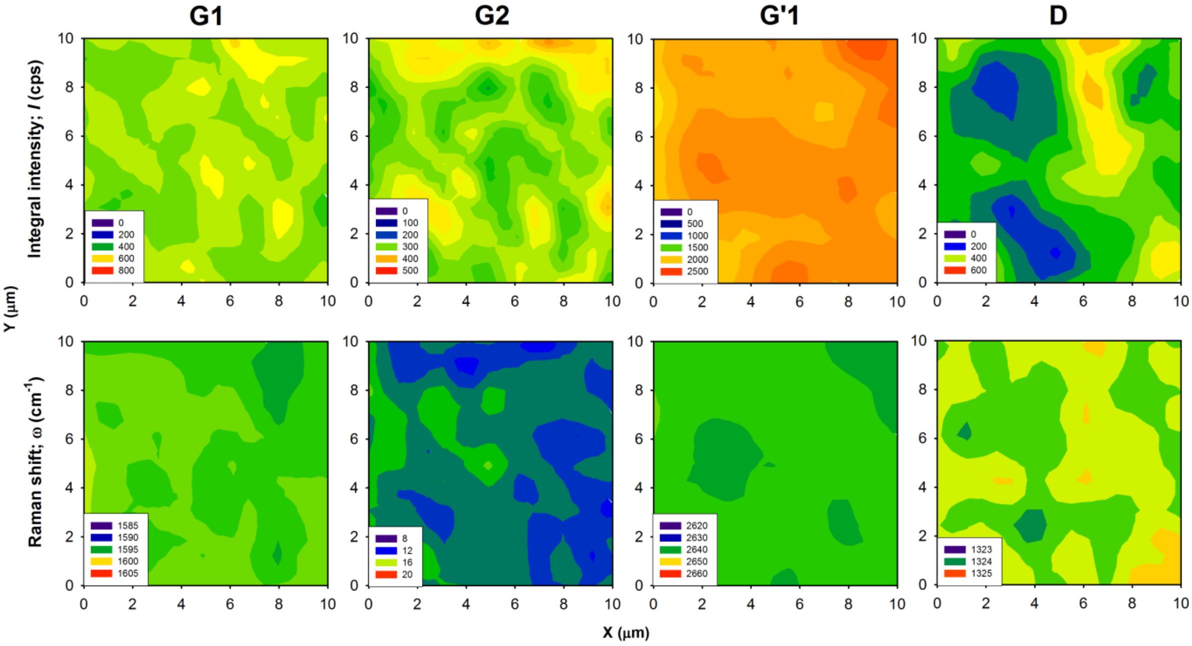

S1.4. Raman maps of Raman shift and integral intensity of the principal graphene modes for the GNP3 sample.

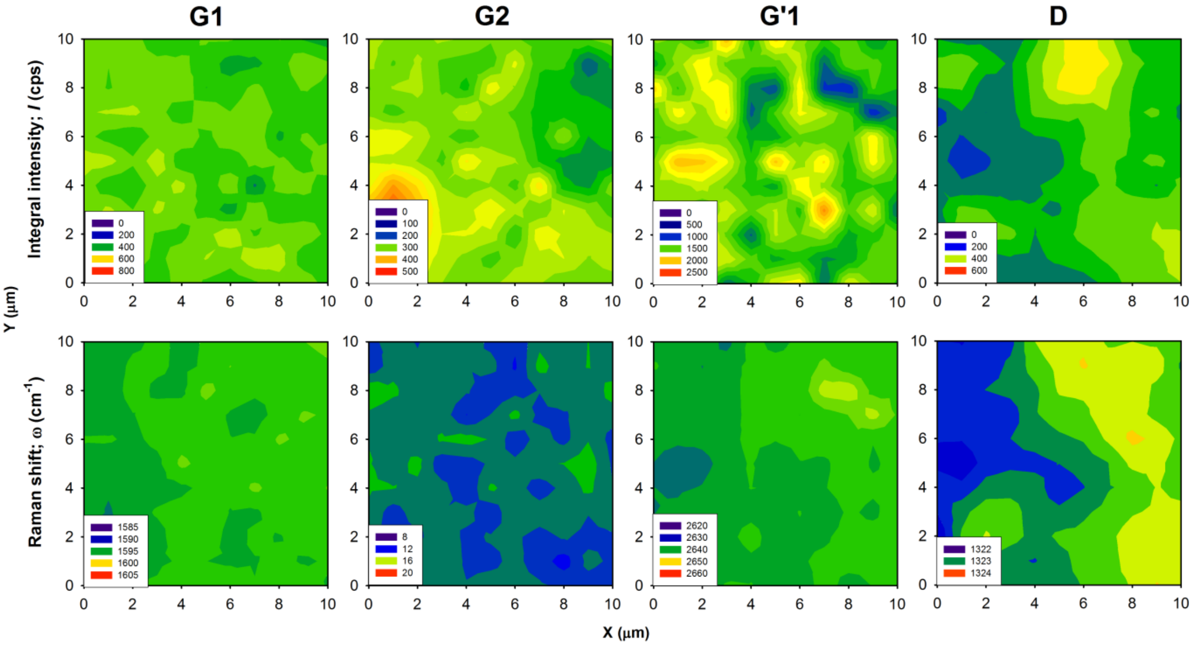

S1.5. Raman maps of Raman shift and integral intensity of the principal graphene modes for the GNP4 sample.

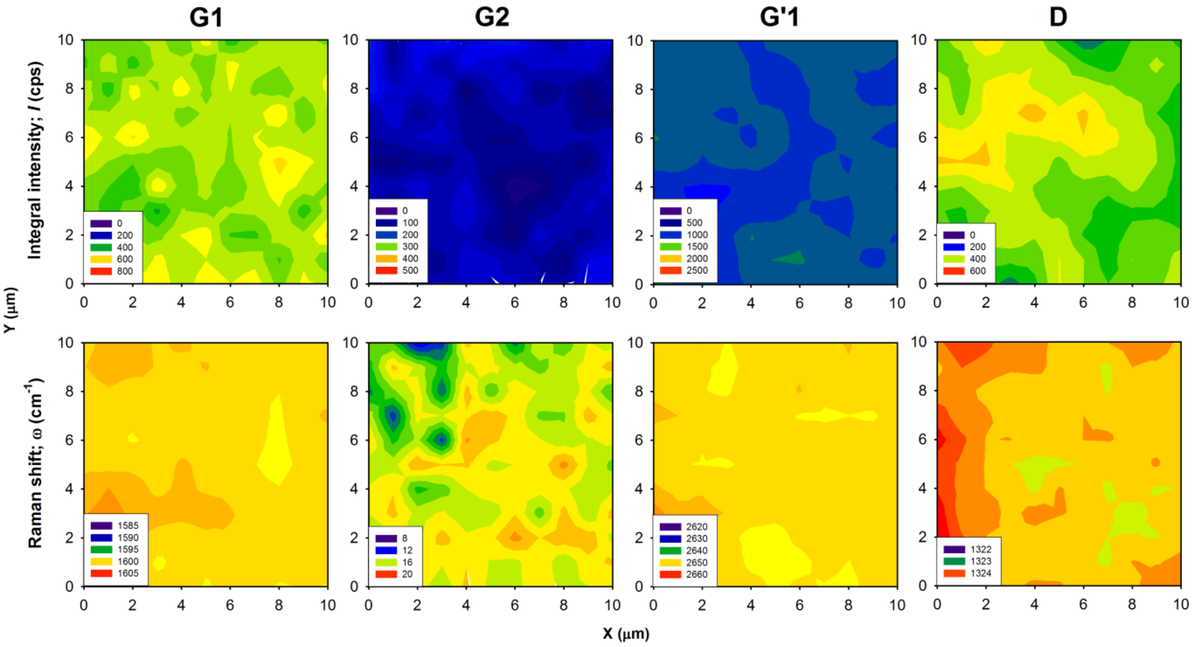

S1.6. Raman maps of Raman shift and integral intensity of the principal graphene modes for the GNP6 sample.

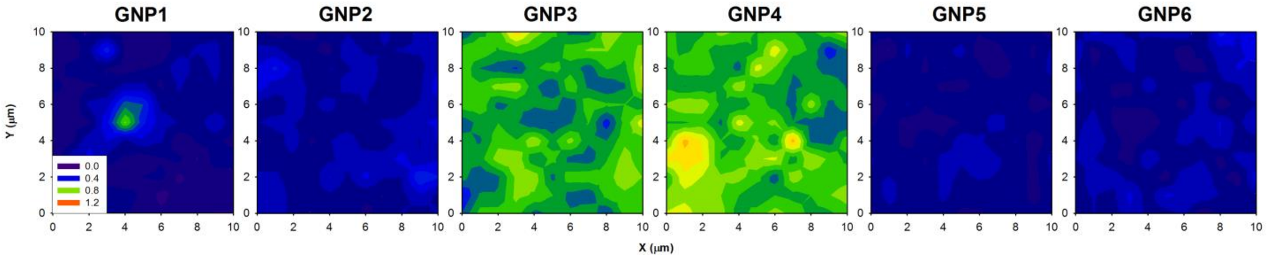

S1.7. Spatial distribution of the relative intensity of the G_2 with respect to the G_1 mode for GNP1 – GNP6 samples.

Table S1.1. Basic parameters obtained from analysis of the fine structure of the G and G' mode: FWHM – full width at half maxima and α - fraction of the Lorentzian component. For the GNP3, the best match of the G' was achieved for a single pseudo-Voigt peak with larger FWHM, therefore the α G'_2 is just estimation from the less significant fit.

Sample	FWHM G_1 (cm^{-1})	FWHM G_2 (cm^{-1})	α G_1	FWHM G'_1 (cm^{-1})	α G'_1	α G'_2
**GNP1**	14.8±0.8	15.0±0.2	0.69±0.11	39.5±1.5	0.60±0.04	0.57±0.32
**GNP2**	14.8±0.6	15.0±0.2	0.53±0.10	43.0±1.8	0.51±0.07	0.77±0.23
**GNP3**	15.2±0.4	15.0±0.2	0.68±0.22	45.4±1.0	0.42±0.05	0.50±0.20*
**GNP4**	15.9±0.4	15.0±0.2	0.43±0.16	44.0±1.4	0.35±0.05	0.98±0.02
**GNP5**	13.6±0.4	15.0±0.2	0.65±0.22	42.6±1.0	0.53±0.06	0.78±0.20
**GNP6**	15.2±0.4	15.0±0.2	0.68±0.22	42.4±1.2	0.49±0.04	0.66±0.18

S.2. Magnetic characterization of the nanoparticles

We performed basic characterization of magnetic properties of the dried NP sample. The temperature dependence of the zero-field-cooled and field-cooled magnetization show characteristic saturation of the FC curve, typical for strongly interacting system of superparamagnetic NPs. Further, the refinement of un-hysteretic loops was carried out in order to determine the median magnetic moment and the so-called magnetic size of the NPs. The mean size fraction corresponds to app. 10 nm large NPs, which agrees well with the values obtained from AFM and SEM and hence suggests excellent crystallinity of the NPs.

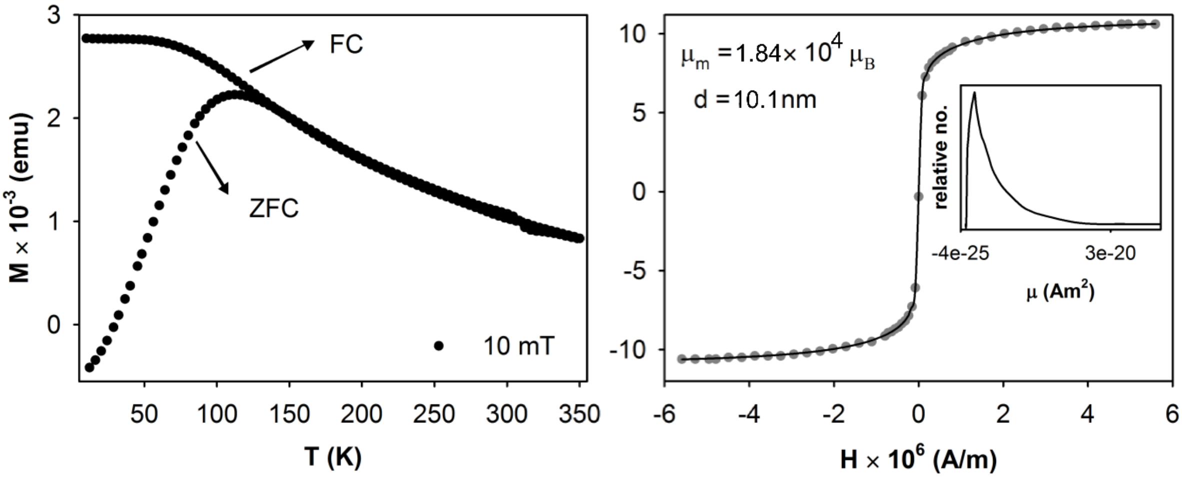

S2.1. Temperature dependencies of ZFC and FC magnetization of the NP sample, together with refinement of the un-hysteretic curves in the SPM state. Distribution of magnetic moments is shown in the inset. Values of the mean magnetic moments and magnetic diameter are also depicted in the image

S.3. Additional characterization of the nanoparticles and GNP1-GNP6 samples by HR SEM and AFM

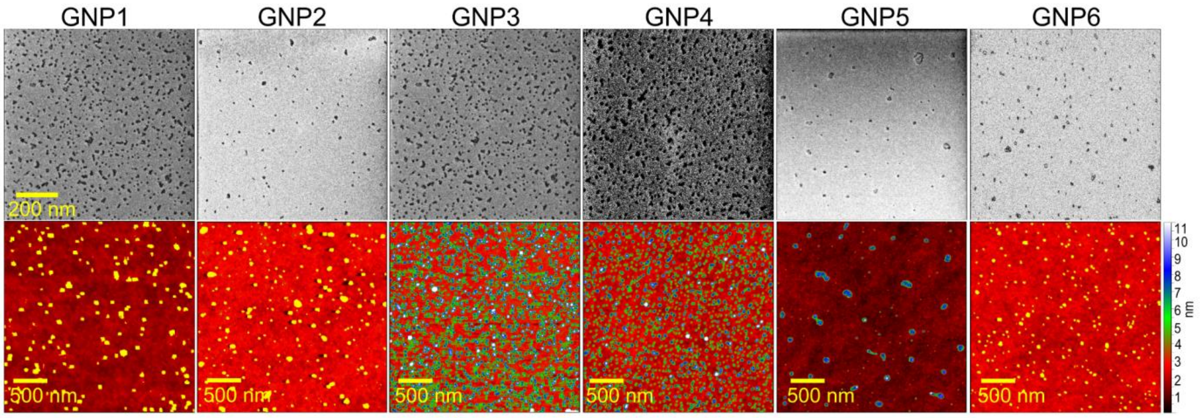

S3.1. High-resolution SEM images of the nanoparticles dispersed on Si/SiO_2 substrate (top) and example of AFM images of the substrate Si/SiO_2 decorated with NPs (bottom).

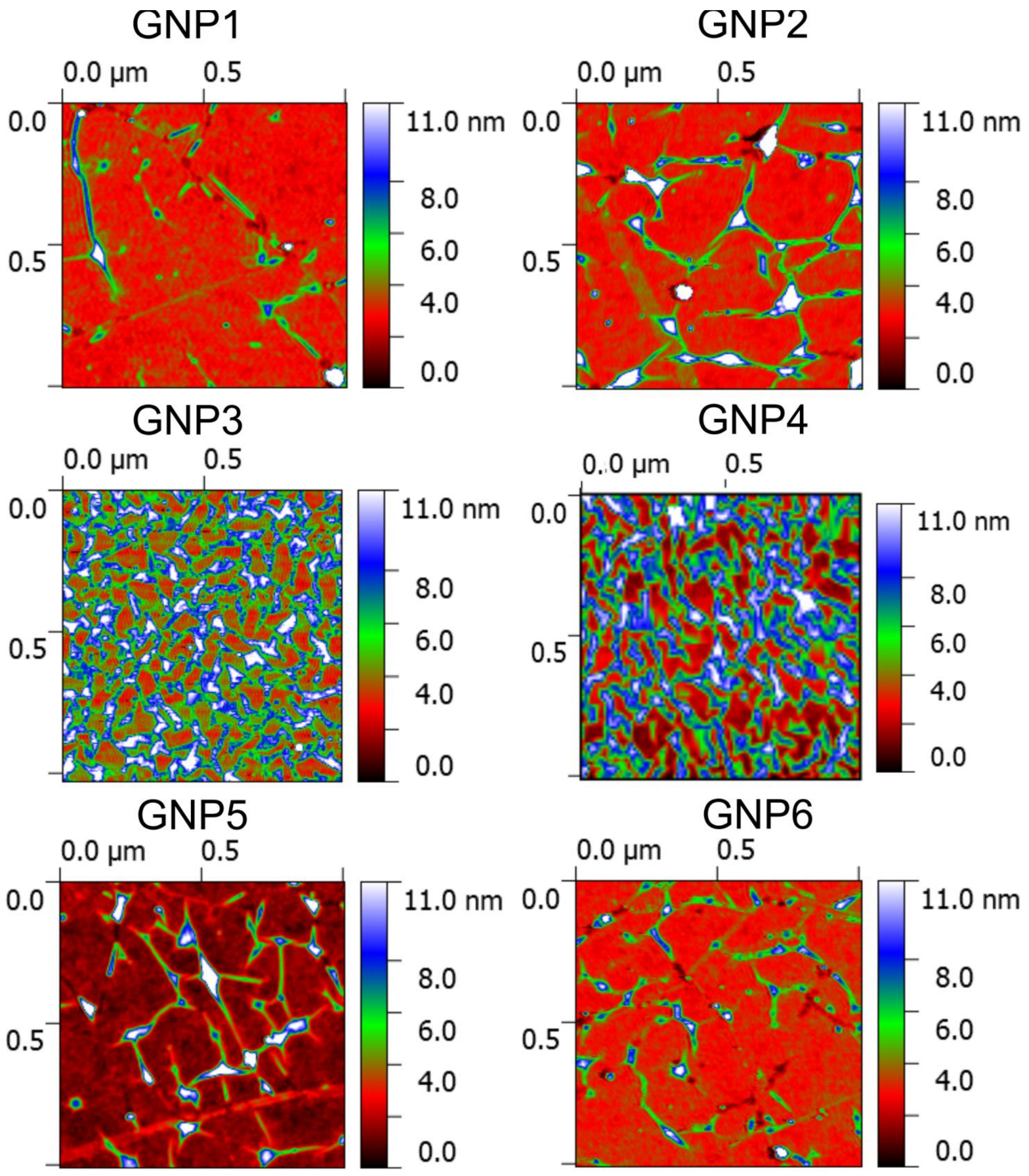

S3.2 High magnification AFM images of the samples GNP1-6.

Table S3.1. Basic parameters obtained from analysis of the HR SEM and AFM imaging: nanoparticle density, N_{NPs}; mean interparticle distance, $d_{NP\text{-}NP}$; wrinkled area of the 1-LG, A_w.

Sample	$N_{NPs}/\mu m^2$	$d_{NP\text{-}NP}$ (nm)	A_w (%)
**GNP1**	77±5	146±5	6±2
**GNP2**	77±5	140±5	14±3
**GNP3**	454±45	54±4	50±5
**GNP4**	438±44	54±4	44±5
**GNP5**	20±3	268±2	10±3
**GNP6**	134±8	104±4	18±3

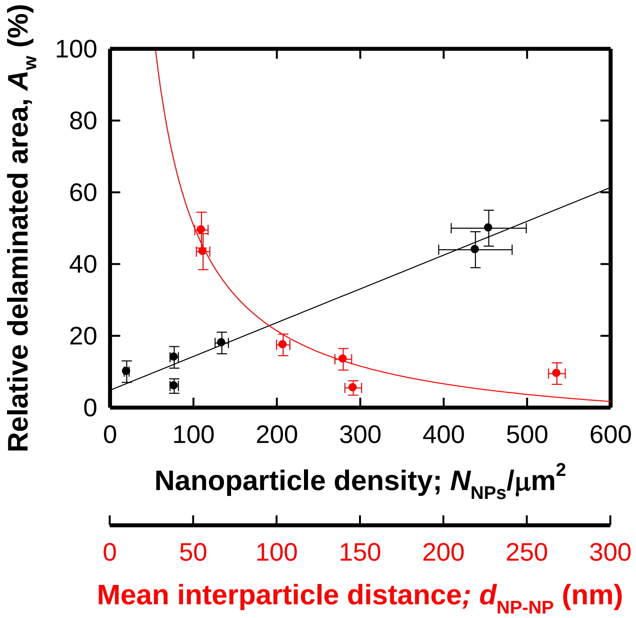

S3.3. Correlation of the mean nanoparticle density, N_{NPs} (black) and mean interparticle distance, d_{NP-NP} (red) to relative delaminated area of 1-LG, A_w determined from AFM data. The $A_w(N_{NPs})$ dependence can be expressed as a linear function: $A_w(N_{NPs}) = a(N_{NPs}) + b$, where a = 0.094±0.009 and b = 4.8±2.4; the $A_w(d_{NP-NP})$ dependence follows approximately a hyperbolic function: $A_w(d_{NP-NP}) = c/(d_{NP-NP}) + d$, where c = 2964±389 and d = 7.6±4.6.

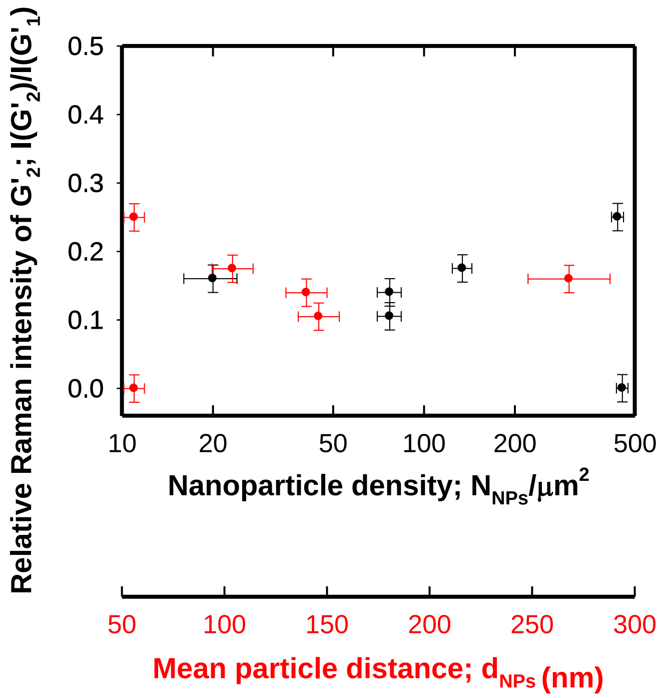

S3.4. Correlation of the key parameters representing the spatial distribution of nanoparticles and level of wrinkling of the 1-LG layer estimated from the analysis of the G' mode (mean nanoparticle density, N_{NPs} (and mean interparticle distance, $d_{\mathrm{NP\text{-}NP}}$ (red), relative area of wrinkles, A_{w}). The dependencies do not show a clear monotonic trend as in case of the G mode-related features due to complex structure of the G' mode.

S.4 Profile analysis of the Raman spectra

The individual Raman peaks were fitted by the profile function Ω(I,ω) (eq.S1) aproximated in the form of the pseudo-Voigt function (linear combination of the Gaussian and Lorentizan as a sufficient approximation of their convolution – Voigt function).The symbols used in equation S4.1 have the following meaning: *I* - Raman intensity, ω - Raman shift, ω_0 - peak position, Γ - full width at half maximum of the peak and α - fraction of the Lorentzian component. The Gaussian component serves as a measure of distribution of the peak parameters due to finite size of the laser spot (~1 μm^2), which is expected to be about one order larger then the local variation of the parameters at nm scale.

$$\Omega(I,\omega) = (1-\alpha) I \sqrt{\ln 2 / 4\pi\Gamma^2} \exp\left[\frac{-\ln 2(\omega-\omega_0)^2}{4\Gamma^2}\right] + \alpha\left[\frac{1}{2\pi\Gamma}\frac{I}{1+\frac{(\omega-\omega_0)^2}{4\Gamma^2}}\right] \qquad \text{(S4.1)}$$
